# New cavernicolous ground beetles from Anhui Province, China (Coleoptera, Carabidae, Trechini, Platynini)

**DOI:** 10.3897/zookeys.923.47322

**Published:** 2020-04-01

**Authors:** Jie Fang, Wenbo Li, Xinhui Wang, Mingyi Tian

**Affiliations:** 1 School of Life Science, Anhui University, Hefei, 230601, China Anhui University Hefei China; 2 Wuhua Branch, Guangdong Tobacco, Shuizhai, 514400, Guangdong, China Unaffiliated Shuizhai China; 3 Department of Entomology, College of Agriculture, South China Agricultural University, 483 Wushan Road, Guangzhou, 510642, China South China Agricultural University Guangzhou China

**Keywords:** carabids, hypogean, new genus, new species, subterranean

## Abstract

A new genus and three new species of cave-adapted ground beetles are reported from the limestone cave Shenxian Dong in Huangshan Shi, southeastern Anhui Province, China. *Shenoblemus***gen. nov.** is proposed to place the anophthalmic trechine species *S. minusculus***sp. nov**. This genus is characterized by the tiny but stout body, sub-moniliform antennae, serrated elytral margins near the base, and a wide distance between the fifth and sixth pores of the marginal umbilicate series on the elytra. In addition, two new species, *Wanoblemus huangshanicus***sp. nov**. (an anophthalmic trechine) and *Jujiroa inexpectata***sp. nov**. (a microphthalmic platynine), are also described and illustrated from the same cave.

## Introduction

The first report of the subterranean ground beetles from Anhui Province, eastern China, was published a few years ago (Fang et al. 2016). In that paper one new genus and two new species of anophthalmic trechines were described: *Wanoblemus wui* Tian & Fang, 2016 from the cave Baiyun Dong in Xuancheng Shi and Cimmeritodes (Zhecimmerites) parvus Tian & Li, 2016 from several caves in Chaohu Shi.

In April 2016, one of us (LWB) conducted cave biodiversity investigations in Tongling, Chizhou, Xuancheng, and Huangshan prefectures of Anhui. His efforts were in vain in four of the five explored caves except Shenxian Dong in Huangshan Shi where he achieved success in collecting seven cave-adapted trechine beetles. Further studies in the laboratory confirmed that six of these beetles belonged to a new species of the genus *Wanoblemus* Tian & Fang, 2016. However, the most interesting discovery was the other single male specimen. It is a representative of a new genus of the *Trechoblemus* phyletic series judging from its small and stout body, together with several other peculiar characteristics.

In order to collect more material, we re-visited the cave Shenxian Dong in late 2018 and April 2019 respectively. Unfortunately, we did not find any more specimen of the latter species. However, we collected some more individuals of the *Wanoblemus* species, and, unexpectedly, we discovered a new hypogean Platynini of the genus *Jujiroa* Uéno, 1952.

We herein provide the description of the new genus and describe the aforementioned three new cave-adapted ground beetles.

## Material and methods

The material was collected by using an aspirator inside the cave Shenxian Dong, and kept in 55% ethanol before study, except one of each species was kept in 95% ethanol for molecular analysis if two or more specimens were available. Dissections and observations were made under a Leica S8AP0 microscope. Dissected male genital pieces, including the median lobe and parameres of the aedeagus, were preserved in Euparal Mounting Medium (BioQuip Products, Inc., CA, USA) onto small transparent plastic plates and pinned under the specimen. Habitus pictures were taken by means of a Keyence VHX-5000 digital microscope. Genital pictures were taken using a Canon EOS 40D camera connected to a Zeiss AX10 microscope. Female genitalia were dissected before the entire abdomen was removed and placed in cold 10% KOH for one day, then cleaned in lactic acid for one day, and stained in Chlorazol Black which was dissolved in 70% ethanol for thirty seconds. All pictures were processed using Adobe Photoshop CS5 computer software.

Measurements and terminology follow Tian et al. (2016). Terminology for female reproductive tract follows Deuve (1993, 2018) and Liebherr and Will (1998).

## Taxonomy

### Shenoblemus

Taxon classificationAnimaliaColeopteraCarabidae

Tian & Fang gen. nov.

55D9F836-7A63-56CE-B246-F80101B2E061

http://zoobank.org/338E5C04-310D-4737-84D5-8697E6899BCB

#### Type species.

*Shenoblemus minusculus* Tian & Fang, sp. nov. (the cave Shenxian Dong, Huangshan Qu, Huangshan Shi, Anhui).

#### Generic characteristics.

Small-sized beetles for the phyletic series of *Trechoblemus* (Casale and Laneyrie 1982; Jeannel 1928; Casale et al. 1998), or *Trechoblemus* complex (Uéno and Pawlowski 1981), anophthalmic; body short and stout, appendages short; dorsal surface more or less pubescent; head subquadrate, wider than long excluding mandibles, and shorter than pronotum; frontal furrows entire, two pairs of supra-orbital and pair of suborbital pores present; right mandible bidentate; labial suture absent, making mentum and submentum completely fused; mentum bisetose, base largely concave, median tooth simple, short and blunt at apex; submentum quadrisetose; antennae short, extending only to about 1/3 of elytra from base, the 7^th^ to 11^th^ antennomeres sub-moniliform; pronotum quadrate, transverse, evidently wider than long, widest near front, at about 1/4 apically, two pairs of lateromarginal setae present, posterior ones located before hind angles, fore angles markedly protruding and sharp, hind ones nearly rectangular and pointed, base nearly straight; elytra stout though distinctly longer than fore body including mandibles, nearly parallel-sided, widest at about middle, surface moderately convex, shoulders distinct, angularly rounded, lateral margins strongly serrated at base, then more or less ciliated throughout; striae obliterated though partly traceable; two dorsal pores present on the 3^rd^ stria, and the preapical present; apical striole weakly defined, connected to the 5^th^ stria; humeral group (the 1^st^ to 4^th^ pores) of marginal umbilicate series equidistantly spaced, median group (the 5^th^ and 6^th^ pores) widely separated each other, the 5^th^ pore forwardly shifted and closer to the 4^th^ than to 6^th^; protibia without longitudinal groove externally; the 1^st^ and 2^nd^ protarsomeres modified in male, distinctly denticulate inwards at each apex; the 1^st^ protarsomere shorter than 2^nd^ to 4^th^ ones combined in all legs; ventrite VII with one pairs of apical setae in male; male genitalia thin and slender, slightly arcuate.

#### Remarks.

The main characteristics (such as the small and pubescent body, two frontal pores present on the head, fused mentum and submentum, location of dorsal pores on the 3^rd^ stria and an equidistantly spaced humeral group of the marginal umbilicate series) indicate that *Shenoblemus* is a lineage of the *Trechoblemus* phyletic series. It is probably close to the Zhejiangese genus *Microblemus* Uéno, 2007, whose members are also small-sized, with similar head, bidentate right mandible, and have similar chaetotaxy on the elytra (Uéno 2007a). However, *Shenoblemus* can be easily distinguished from *Microblemus* by: (1) antennae sub-moniliform, whereas they are filiform in *Microblemus*; (2) mentum and submentum completely fused, versus only partly fused with labial suture traceable in *Microblemus*; (3) pronotum quadrate and transverse, with fore angles evidently protruding, but cordate and narrow, with fore angles not protruding in *Microblemus*; and (4) elytral base and shoulders simple or moderately serrate in *Shenoblemus*, whereas they are strongly dentate in *Microblemus*.

*Shenoblemus* may be also related to the sympatric *Wanoblemus* as both genera share some important characteristics: (1) similar chaetotaxy on head and pronotum; (2) 1^st^ and 2^nd^ protarsomeres modified in the male; (3) labial suture missing; and (4) bidentate right mandible (though tricuspid in three individuals of *Wanoblemus huangshanicus* sp. nov.). However, they are evidently different in the following aspects: (1) body much smaller and stouter in *Shenoblemus*; (2) 7^th^ to 11^th^ antennomeres are sub-moniliform in *Shenoblemus*, but filiform in *Wanoblemus*; (3) pronotum strongly transverse, with fore angles protruding and sharp in *Shenoblemus*, versus narrower, with fore angles not protruding in *Wanoblemus*; (4) lateral margin of elytra strongly serrate near base in *Shenoblemus*, whereas it is weakly subserrate or ciliate in *Wanoblemus*; (5) 5^th^ and 6^th^ pores of the marginal umbilicate series of the elytra widely spaced, making the 5^th^ pore closer to the 4^th^ than to the 6^th^ in *Shenoblemus*, instead of much closer to the 6^th^ than to the 4^th^ in *Wanoblemus*; (6) protibiae without a longitudinal sulcus in *Shenoblemus*, versus a distinct longitudinal sulcus present in *Wanoblemus*; and (7) male genitalia thin and elongate in *Shenoblemus*, versus short and strongly arcuate in *Wanoblemus*.

The following features may separate *Shenoblemus* from another Zhejiangese genus, *Wulongoblemus* Uéno, 2007 whose members have also the 1^st^ and 2^nd^ protarsomeres modified in the male: (1) right mandible bidentate in *Shenoblemus*, but tridentate in *Wulongoblemus*; (2) body small, short, and stout in *Shenoblemus*, whereas it is large and slender in *Wulongoblemus*; (3) pronotum transverse, with fore angles protruding in *Shenoblemus*, versus pronotum longer than wide, with obtuse fore angles in *Wulongoblemus*; (4) antennae sub-moniliform in *Shenoblemus*, instead of filiform in *Wulongoblemus*.

#### Etymology.

“Shen (= “Shenxian”, meaning immortal in Chinese) + blemus”, referring to the locality of the type species. Gender masculine.

#### Generic range.

China (Anhui).

### Shenoblemus minusculus

Taxon classificationAnimaliaColeopteraCarabidae

Tian & Fang sp. nov.

D9F5FA61-A750-586E-8CA0-B9915AFFA07A

http://zoobank.org/2888D9C3-1870-4244-B846-8EBBB711BC0F

**Figure 1.**
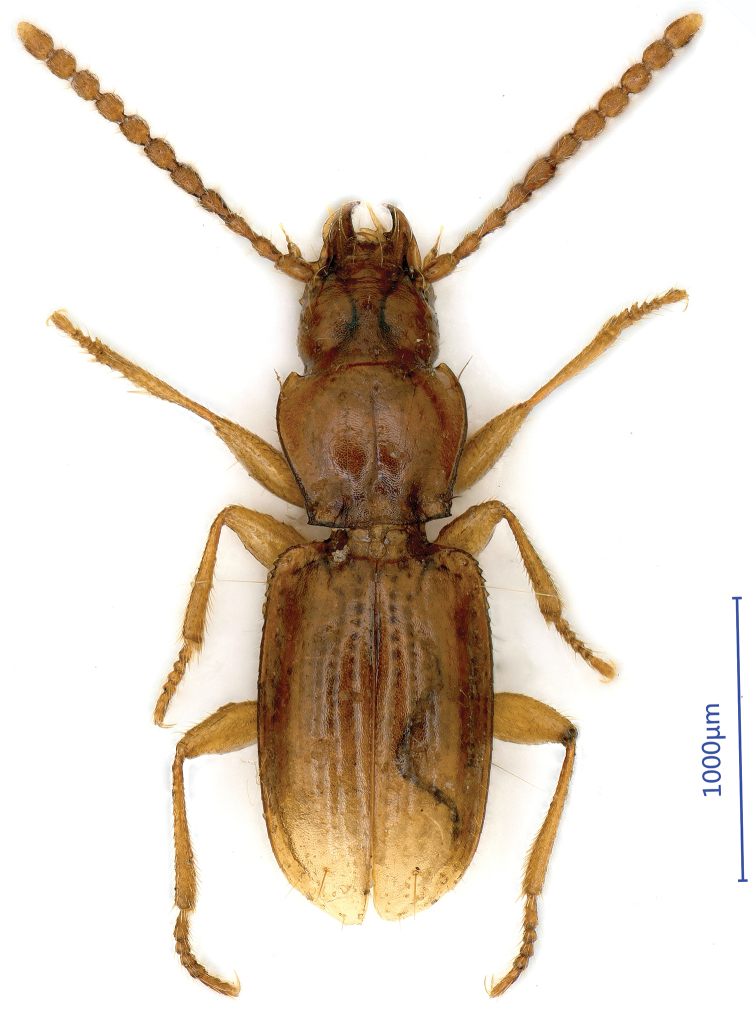
Habitus of *Shenoblemus minusculus*, sp. et gen. nov., male, holotype.

**Figure 2.**
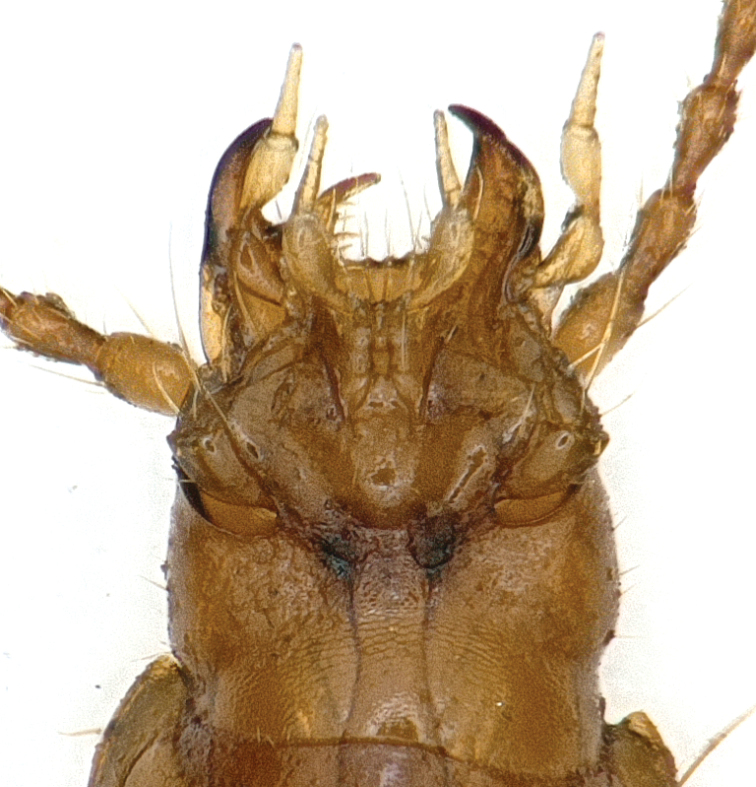
Head of *Shenoblemus minusculus*, sp. et gen. nov., male, holotype.

**Figure 3.**
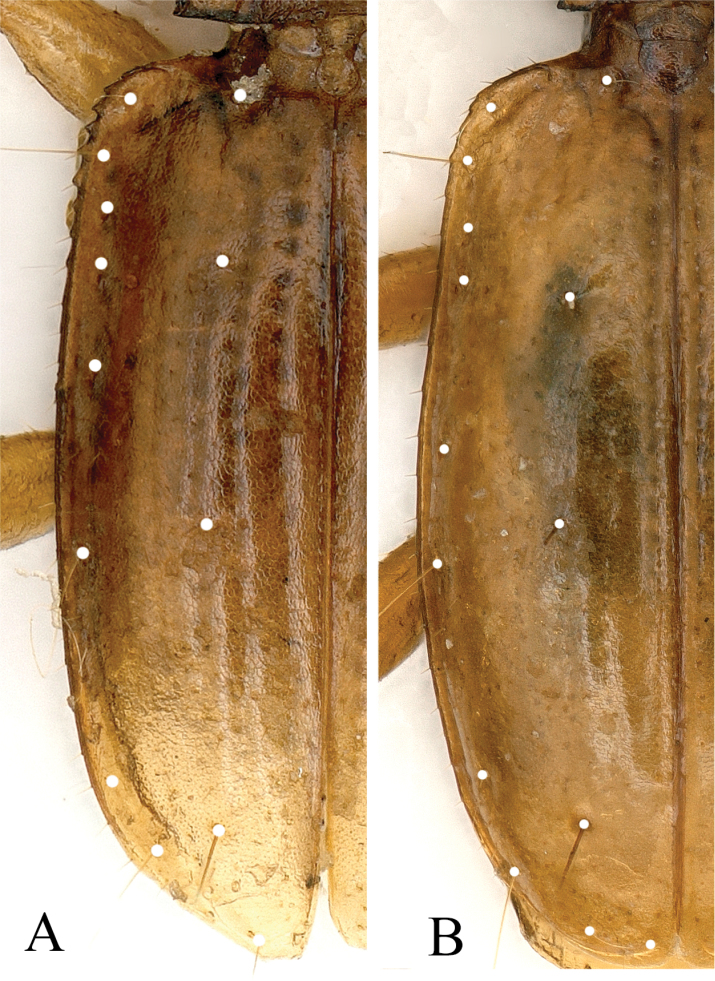
Left elytra of two cave trechines to show chaetotaxy **A***Shenoblemus minusculus*, sp. et gen. nov., male, holotype **B***Wanoblemus huangshanicus*, sp. nov., male, holotype.

**Figure 4.**
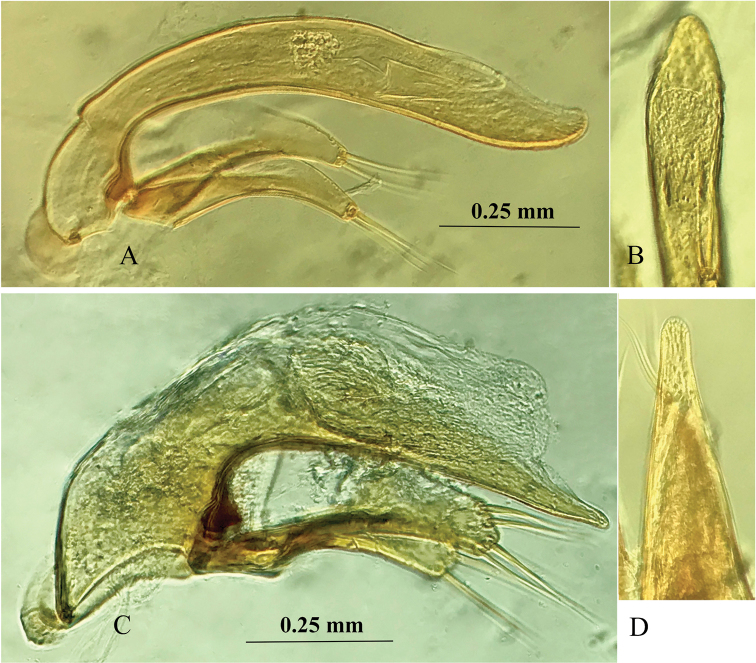
Male genitalia of *Shenoblemus minusculus*, sp. et gen. nov. (**A, B**) and *Wanoblemus huangshanicus*, sp. nov. (**C, D**) **A, C** median lobe and parameres, lateral view **B, D** apical lobe, dorsal view.

Figs 1 , 2 , 3A , 4A, B

#### Material.

***Holotype*** male, cave Shenxian Dong, Qiaoshan, Xinming, Huangshan, Anhui, 30°23'9.55"N, 118°14'7.66"E, 366 m in altitude, 2016-IV-22, leg. Wenbo Li, deposited in the insect collections of South China Agricultural University, Guangzhou, China (SCAU).

#### Diagnosis.

Small-sized, eyeless and yellowish-brown beetle, with stout body and short appendages; covered with pubescence which are sparser on head, prothorax and elytra, and denser on abdominal ventrites.

#### Description.

***Length***: 2.45 mm; width: 0.84 mm. Habitus as in Fig. 1. ***Fore body*** (head plus pronotum including mandibles) much shorter than elytra. Microsculpture made of isodiametric meshes irregularly distributed on head, pronotum and elytra.

***Head*** large and widened, a little narrower than head including mandibles, HW/HLm = 0.90, or wider excluding mandibles, HW/HLl = 1.17; widest at about 1/4 of head from base excluding mandibles; front and vertex convex; frontal furrows well-defined and complete, strongly divergent at both anteriorly and posteriorly; genae markedly expanded laterally; anterior and posterior supra-orbital pores close, both at widest area of genae; clypeus quadrisetose, labrum transverse, almost straight in the front margin, 6-setose; mandibles widened and moderately curved at apices; ligula thin and short, adnated with paraglossae; palps short, penultimate joints much stouter than apical ones; the 2^nd^ labial palpomere slightly longer than the 3^rd^, bisetose on inner margin, with two or three additional setae at subapex on outer margin; the 3^rd^ maxillary palpomere as long as the 4^th^; the 1^st^–6^th^ antennomeres filiform, while the 7^th^–11^th^ semi-moniliform; relative length of each antennomere as: the 1^st^ (1.13), 2^nd^ (1.00), 3^rd^ (1.08), 4^th^ (1.04), 5^th^ (1.13), 6^th^ (1.00), 7^th^ (1.04), 8^th^ (1.04), 9^th^ (1.04), 10^th^ (1.00) and 11^th^ (1.42).

***Pronotum*** moderately transverse, PnW/PnL = 1.21, as long as head (including mandibles), much wider than head, PnW/HW = 1.32; lateral margins finely bordered, anterior lateromarginal pores located at about apical sixth, posterior ones at a little before hind angles; base slightly narrower than front, PbW/PfW = 0.97, both nearly straight and unbordered; hind angles denticulate at tips; both frontal and basal impression faint, basal foveae large and deep; disc moderately convex. Scutellum small and almost rounded.

***Elytra*** much longer than wide, EL/EW = 1.56, wider than pronotum, EW/PnW =1.24; unbordered at base; strial punctures large and widely isolated; basal pores distant from scutellum, anterior and posterior dorsal pores on the 3^rd^ striae at about 1/4 and 4/7 of the elytra from the base respectively; preapical pores at about apical 1/7 of elytra, subequal to suture and apical margin; humeral group of marginal umbilicate series regular though the 2^nd^ closer to marginal gutter than other; the 5^th^ widely separated from the 6^th^, apical pores close to elytral margin (Fig. 3A).

***Legs*** short and stout, densely pubescent; protarsi short, the 1^st^ tarsomere much shorter than the 2^nd^–4^th^ combined in fore and middle legs, whereas slightly shorter in hind legs; abdominal ventrites IV - VI each with a pair of paramedical setae in male.

***Male genitalia*** (Fig. 4A, B): The median lobe of aedeagus moderately sclerotized, rather long and thin, weakly arcuate at median portion, rounded at apex; dorsal margin suddenly folded at about basal quarter; base small, sagittal aileron quite large and hyaline, inner sac provided with a triangular and small copulatory piece, which is about 1/5 as long as aedeagus; in dorsal view, apical lobe widened at subapex, nearly triangular form, longer than wide, with a broadly rounded apex; parameres moderately elongated, right one as long as the left, each armed with two long setae at apex.

**Female**: Unknown.

#### Etymology.

To refer to the small body size.

#### Distribution.

China (Anhui). Known only from a limestone cave called Shenxian Dong in Huangshan Shi.

The Shenxian Dong limestone cave is more than 3000 m long. It is divided in three parts (front, middle, and back) for touristic purpose. There is an underground stream along the main passage. The single beetle specimen was collected in a dark area approximately 50 m from the entrance.

### Wanoblemus huangshanicus

Taxon classificationAnimaliaColeopteraCarabidae

Tian & Li sp. nov.

F57C0544-A895-5086-B048-06C12673A504

http://zoobank.org/2BD1BC1D-89D0-4C8B-94C3-12F0EB77F55F

**Figure 5.**
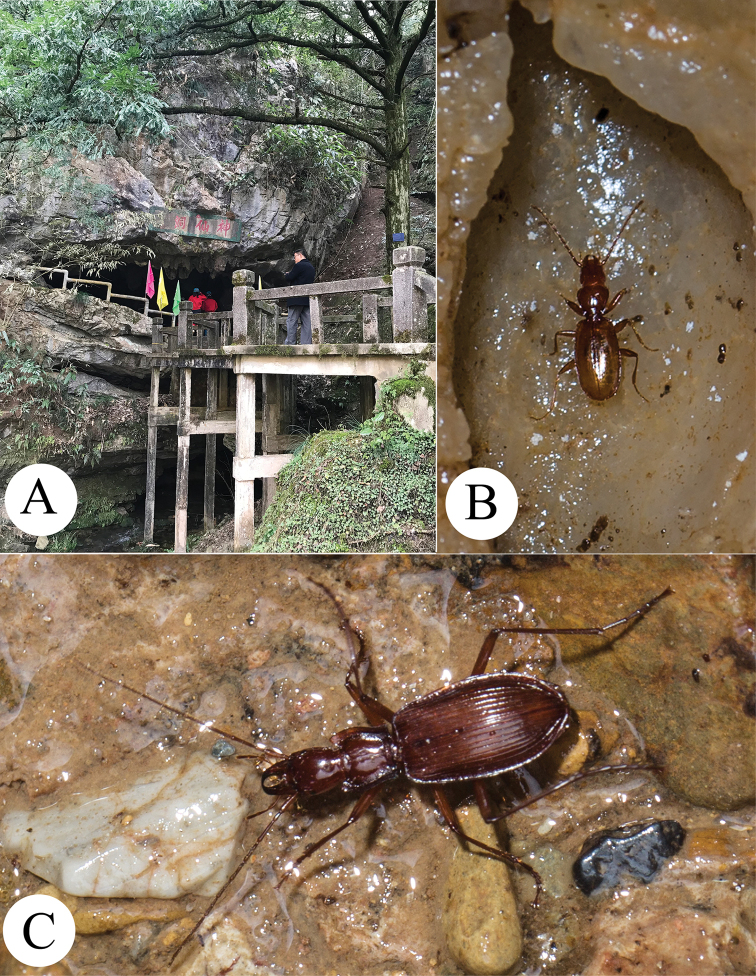
Cave Shenxian Dong, the type locality of three new species **A** entrance **B** a running individual of *Wanoblemus huangshanicus*, sp. nov. **C** a running individual of *Jujiroa inexpectata*, sp. nov.

**Figure 6.**
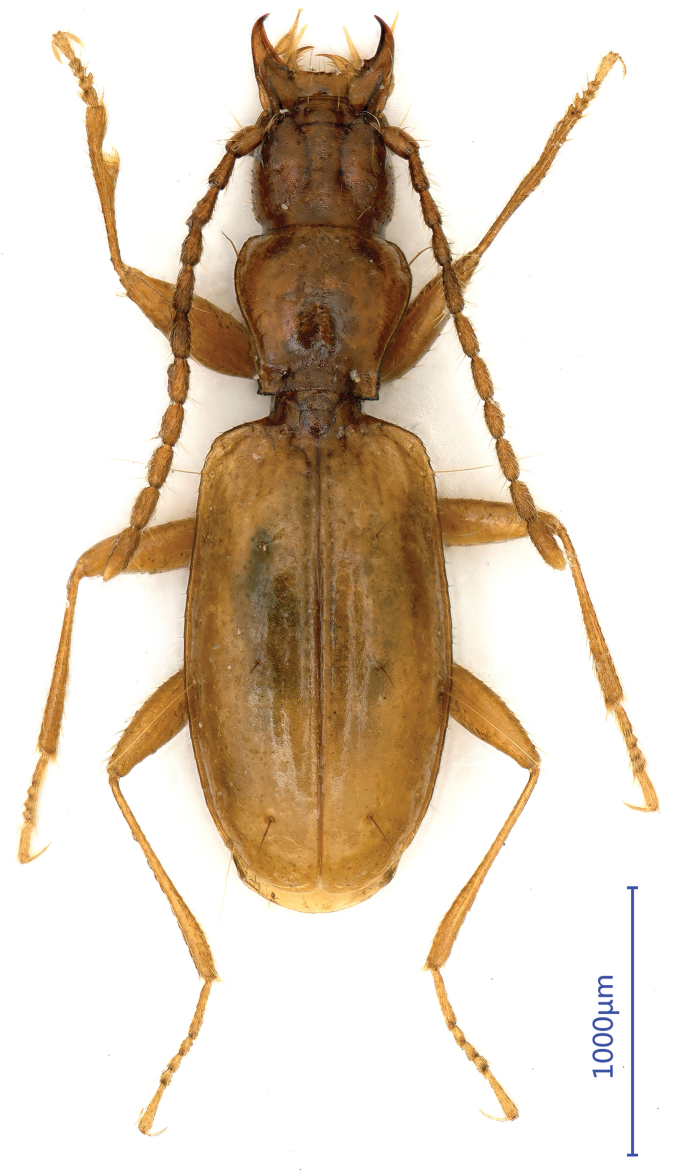
Habitus of *Wanoblemus huangshanicus*, sp. nov., male, holotype.

Figs 3B , 4C, D , 5B , 6

#### Material.

***Holotype***: male, cave Shenxian Dong, Qiaoshan, Xinming, Huangshan, Anhui, 30°23'9.55"N, 118°14'7.66"E, 366 m in altitude, 2016-IV-22, leg. Wenbo Li, in SCAU; ***Paratypes***: 3 males and 2 females, idem; 12 males and 15 females, same cave, 2018-XII-24, leg. Weibo Li, Mengzhen Chen, Zhuanghui Qin, Jingli Cheng & Mingyi Tian, in SCAU and in the animal collections of Anhui University, Hefei, China (AHU) respectively.

#### Diagnosis.

A rather small eyeless trechine beetle, yellowish brown, body thin and elongate, sparsely pubescent.

#### Description.

***Length***: 3.2–3.5 mm (including mandibles); width: 0.9–1.0 mm. Habitus as in Fig. 6.

***Head*** longer than wide, HLm/HW = 1.38–1.42, HLl/HW = 1.08–1.2; right mandible bidentate (but tricuspid in three individuals); antennae extending beyond basal 2/5 of elytra.

***Pronotum*** slightly wider than longer, PnL/PnW = 0.90–0.93, shorter than head, PnL/HLm = 0.72–0.75, wider than head, PnW/HW = 1.20–1.24, base narrower than front, PbW/PfW = 0.83–0.87, lateral margins more contracted behind the widest portion than in *Wanoblemus wui* Tian & Fang, 2016.

***Elytra*** slightly thinner than those in *W. wui*, longer than fore body, EL/(HLm+PnL) = 1.28–1.31, EL/(HLl+PnL) = 1.50–1.62, much longer than wide, EL/EW = 1.77–1.81; much wider than pronotum, EW/PnW = 1.51–1.53; chaetotaxy similar in *W. wui* (Fig. 3B).

VII ventrite bisetose in male, while quadrisetose in female.

***Male genitalia*** (Fig. 4C, D): The median lobe of aedeagus well-sclerotized, small but more elongate than the one of *W. wui*, and less curved ventrally in middle part; membranous opening large, rather sharp at apex, base larger, with a small sagittal aileron; in dorsal view, apical lobe much longer than wide, gently contracted towards apex which is broad; parameres shorter than median lobe, both widened at apices, each armed with four long apical setae.

#### Remarks.

This new species is very similar to the type species *W. wui* which occurs in the cave Baiyun Dong, Xuancheng Shi, approximately 46 km in a straight-line distance from the cave Shenxian Dong. However, the new species is smaller and slenderer than the type species, and with a longer and more elongated aedeagus.

#### Etymology.

To refer to the type locality.

#### Distribution.

China (Anhui). Known only from the cave Shenxian Dong in Huangshan Shi.

The specimens collected in 2016 were found in a small wet area about 300 m from the cave entrance. The specimens collected in 2018 were found in dark areas 200–300 m from the entrance.

### Jujiroa inexpectata

Taxon classificationAnimaliaColeopteraCarabidae

Tian & Wang sp. nov.

4E580716-BAE6-5B27-A800-E31DF713FFD9

http://zoobank.org/6D0B38E5-FF5F-4F31-87D5-2DA3CEBC58C6

**Figure 7.**
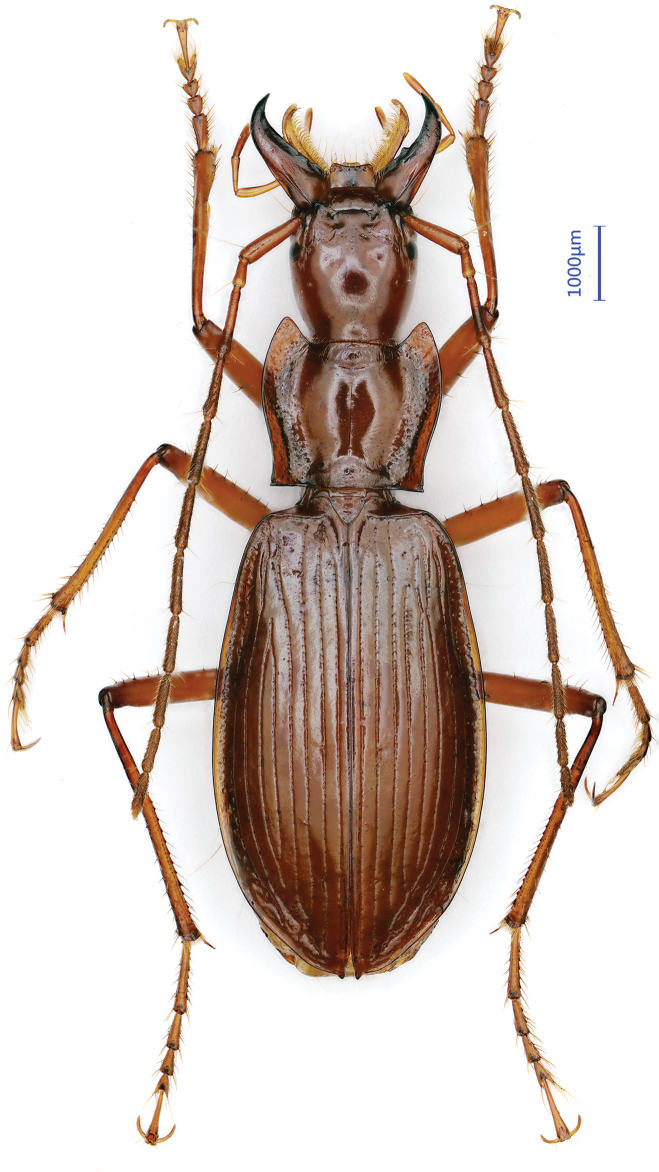
Habitus of *Jujiroa inexpectata*, sp. nov., male, holotype.

**Figure 8.**
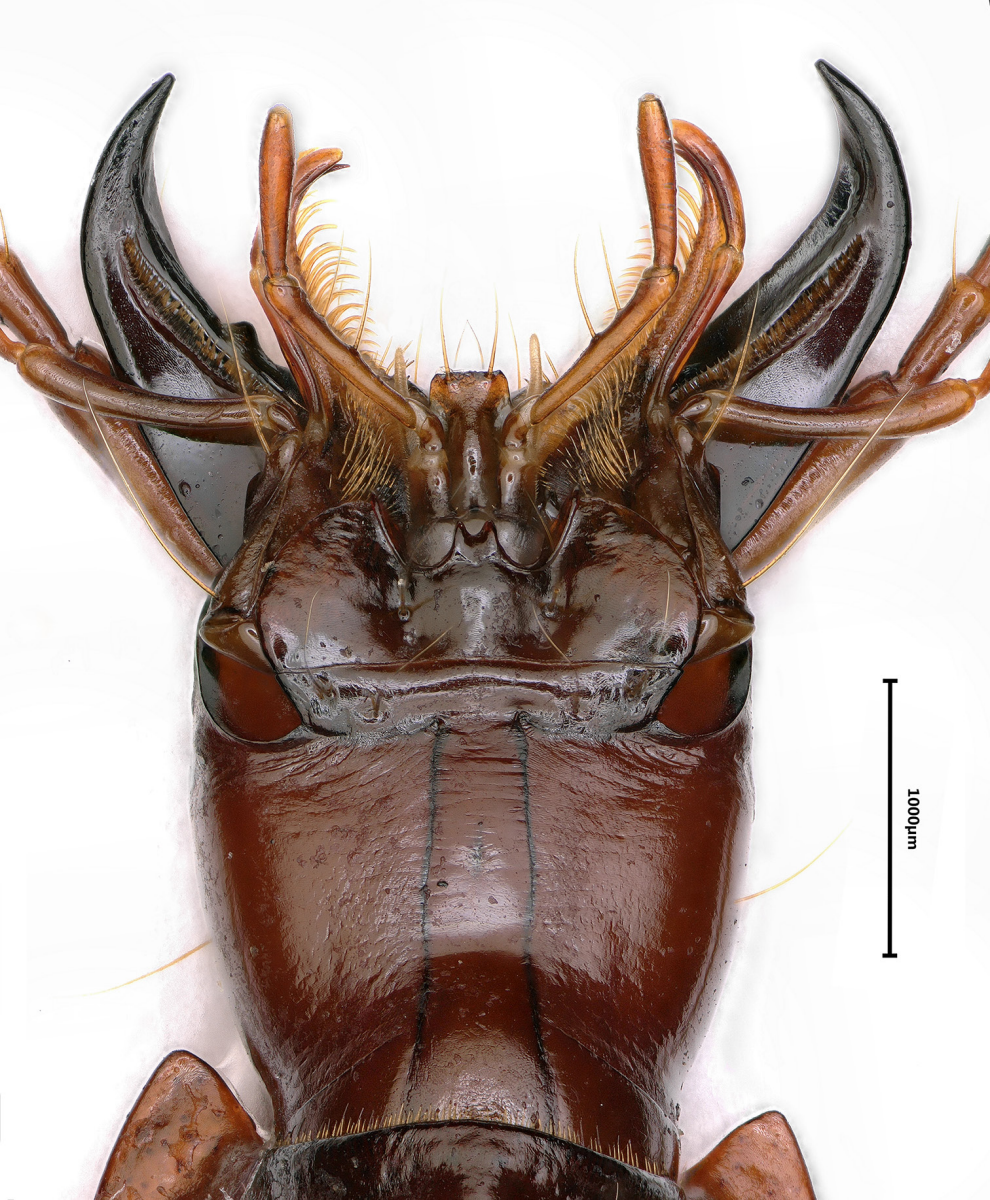
Ventral head of *Jujiroa inexpectata*, sp. nov., male, paratype.

**Figure 9.**
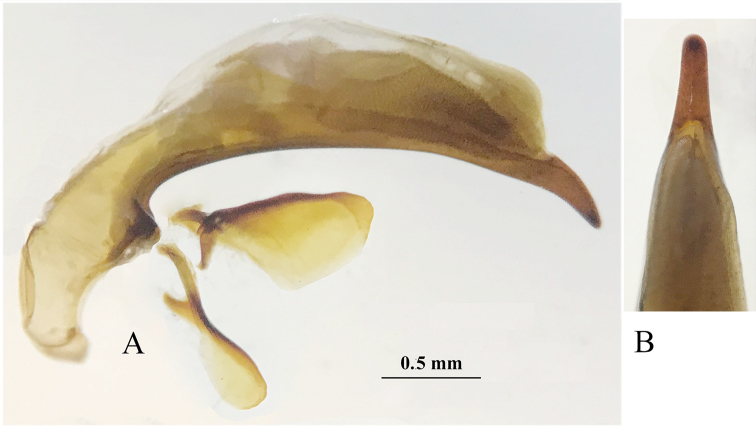
Male genitalia of *Jujiroa inexpectata*, sp. nov., male, holotype.

**Figure 10.**
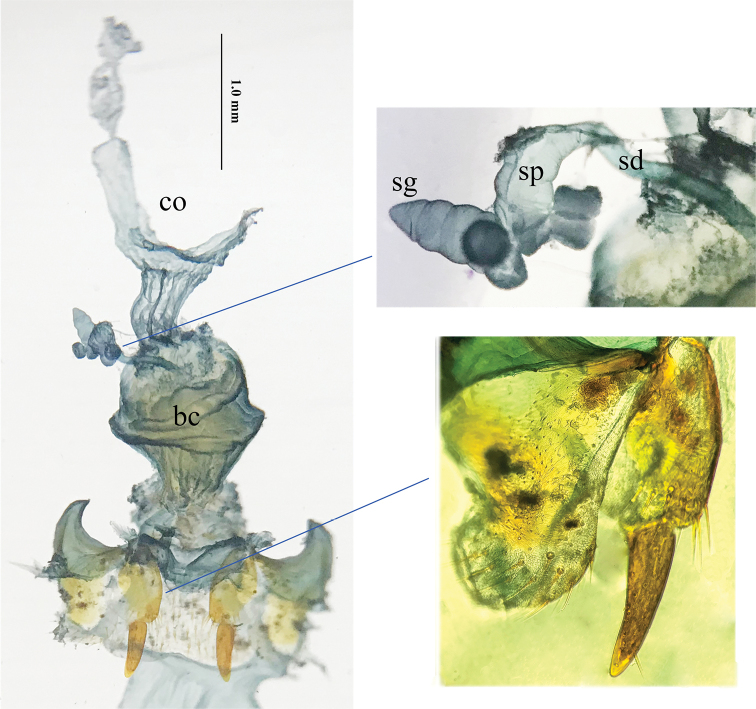
Female reproductive tract of *Jujiroa inexpectata* sp. nov., bc., bursa copulatrix; co., common oviduct; sd., spermathecal duct; sg., spermathecal gland; sp., spermatheca.

Figs 5C , 7 , 8 , 9 , 10

#### Material.

***Holotype***: male, cave Shenxian Dong, Qiaoshan, Xinming, Huangshan, Anhui, 30°23'9.55"N, 118°14'7.66"E, 366 m in altitude, 2018-XII-24, leg. Jingli Cheng, in SCAU; ***Paratypes***: 3 males and 2 females, idem, in SCAU; 2 males, same cave, 2019-IV-12, leg. Ye Liu and Wenbo Li, in National Museum of Zoology, Institute of Zoology, Chinese Academy of Sciences, Beijing (IOZ).

#### Diagnosis.

A medium-sized *Jujiroa* species, body de-pigmented, microphthalmic, pronotum widely reflexed along lateral margins, with strongly protruding fore angles and acute hind angles, elytra with three dorsal setiferous pores on the 3^rd^ intervals and mucronate at apices.

#### Description.

***Length***: 12.5–15.0 mm; width: 4.0–5.0 mm. Habitus as in Fig. 7.

***Body concolorous***, light reddish brown, smooth and glabrous (though sparsely punctate on the reflexed lateral margins of pronotum), strongly shiny. Microsculpture made of nearly isodiametric meshes on front of head, while striate on pronotum and moderately transverse on elytra.

***Head*** ovate (Figs 7, 8), much longer than wide, HLm/HW = 1.8–2.0, HLl/HW = 1.4–1.5; widest at middle of head from base to labrum; genae convex, expanded at sides, frontal furrows short and foveate; two pairs of supra-orbital pores present and nearly on parallel lines; eyes very small, slight convex; clypeus bisetose, labrum emarginate at front; mandibles elongate, teeth evidently reduced; labial suture complete; mentum with two setae on each side just in front of basal pit which was not well-defined; median tooth short, sharply bifid at tip; submentum with two setae on each side, inner ones longer than the outer; ligula short, widened and bisetose at apex; palpomeres slender, the 2^nd^ labial palpomere 1.3 times as long as 3^rd^, the 3^rd^ maxillary palpomere slightly shorter than 4^th^; suborbital setae absent; antennae filiform, extending to about apical 1/4 of elytra, the 1^st^ to 3^rd^ antennomeres glabrous, the 2^nd^ shortest, relative length of each antennomere as: the 1^st^ (2.61), 2^nd^ (1.00), 3^rd^ (2.12), 4^th^ (1.90), 5^th^ (2.00), 6^th^ (1.82), 7^th^ (1.83), 8^th^ (1.61), 9^th^ (1.66), 10^th^ (1.34) and 11^th^ (1.48).

***Pronotum*** transverse, PW/PL = 1.20–1.25; slightly shorter than head excluding mandibles; widest at about 1/3 from front, lateral margins including fore and hind angles widely reflexed throughout, distinctly sinuate before hind angles, only basal lateromarginal setae present at hind angles; fore angles extraordinarily protruding, nearly triangular and bluntly sharpened; hind angles nearly rectangular and pointed; whole margins including base and front without borders, base slightly wider than front including fore angles; both base and front truncate. Scutellum small, inverted triangular.

***Elytra*** elongate, much longer than wide, EL/EW = 1.61–1.83; longer than fore body including mandibles, much wider than pronotum; base well-bordered, shoulders nearly rectangular though rounded; widest at about apical 4/7 of elytra, apex protruding backwardly, mucronate, each elytron with an acute spine, both inner angles evidently divergent; disc slightly convex though largely depressed, striae entire, moderately impressed and punctate; scutellar striole short; basal pores present; interval 3 with three dorsal setiferous pores, at about 1/4, 1/2 and 3/4 of elytra from base, respectively, the anterior close to the 3^rd^ stria, the other two close to the 2^nd^ stria; preapical pore present, at about apical 1/7 of elytra, closer to elytral margin than to suture; two apical pores present; 18 marginal umbilicate pores present throughout.

***Ventral*** surface smooth and glabrous. Legs slender and elongate, procoxa asetose, mesocoxa unisetose, metacoxa bisetose, without inner seta; pro-, and mesotrochanters unisetose, metatrochanters asetose; metafemur bisetose posteriorly in male, trisetose in female; tibiae and tarsi smooth, without longitudinal sulci or striae externally; the 4^th^ tarsomere bilobed in fore and middle legs, whereas deeply emarginated in hind ones. Each abdominal ventrite IV-VI bisetose, ventrite VII bisetose in male, but quadrisetose in female.

***Male genitalia*** (Fig. 9): Similar in *Jujiroa satoi* Uéno, 2007, but slenderer and more elongate, with smaller sagittal aileron, wider basal opening and broader parameres. Median lobe thin and narrow, slightly arcuate in middle portion, then gently curved towards apex with a long and blunt apical lobe. In lateral view, apical lobe thin, much longer than wide.

***Female reproductive tract*** (Fig. 10): Abdominal ventrite X sparsely setose; gonocoxites 1 and 2 similar in other *Jujiroa* species, the former bearing eight fringe setae along apical margin, the latter triangular, slightly curved outwardly, blunt at apex, without lateral or dorsal ensiform setae, but with a tiny seta on outer margin and subapical setose organ; bursa copulatrix wide, simply saccate, with middle part evidently folded, narrowed at base; oviduct situated in middle position, both spermathecal gland and spermatheca twisted, connected each other by a short spermathecal gland duct, spermathecal duct fairly long.

#### Remarks.

The genus *Jujiroa* Uéno, 1952 is known from Japan (Habu 1978, 1981; Takakura 1987; Sasakawa 2006), Vietnam, and from Taiwan Province and mainland China (Jedlička 1961; Casale 1988; Uéno and Saito 1991; Vigna Taglianti 1995, Uéno and Kishimoto 2001; Deuve 2004; Uéno 2007b; Deuve and Pütz 2013). All of the five presently known species from mainland China are cave-adapted beetles: *J. rufescens* (Jedlička, 1961) from Jiangxi; *J. iolandae* Vigna Taglianti, 1995, *J. satoi* Uéno, 2007, *J. deliciola* Uéno & Kishimoto, 2001 and *J. lingguanensis* Deuve et Pütz, 2013 from Sichuan; and *J. clarkei* Deuve, 2004 from Guangxi. These species are usually very rare as all are monotypic species (except *J. satoi*, which was described based on three type specimens) and are known only by the type material. Therefore, we were quite surprised when we collected several individuals together in the cave during a two-hour survey.

Regarding the hypogean *Jujiroa* species from mainland China, this new species is easily separated from *J. clarkei* by the presence of a spinous elytral apex (apical margin of elytra is rounded in *J. clarkei*), from *J. lingguanensis* by its broader body and sharpened hind angles of pronotum, and from the other three species by its smooth tarsomeres which are without longitudinal sulci.

However, *J. inexpectata* sp. nov. is closely similar to *J. iolandae* Vigna Taglianti, 1995, which occurs in Huaying, Sichuan, but it differs by fore angles of pronotum which is more protruding than in *J. iolandae*, by its elytron which is presence of three dorsal setiferous pores, versus anterior pores absent in *J. iolandae*, and by its tarsi which are smooth, whereas longitudinally striated in *J. iolandae*.

#### Etymology.

To indicate that it was a surprise to find this interesting species.

#### Distribution.

China (Anhui). Known only from the cave Shenxian Dong in Huangshan Shi.

## Supplementary Material

XML Treatment for Shenoblemus

XML Treatment for Shenoblemus minusculus

XML Treatment for Wanoblemus huangshanicus

XML Treatment for Jujiroa inexpectata
